# Personalized preventive medicine using genomic information: future perspective and corresponding research plan

**DOI:** 10.3389/fonc.2025.1599753

**Published:** 2025-06-09

**Authors:** Hiroto Narimatsu, Kaname Watanabe, Ann Sato, Eri Haneda, Masumi Okamoto, Haruka Nakada, Sho Nakamura

**Affiliations:** ^1^ Department of Genetic Medicine, Kanagawa Cancer Center, Yokohama, Japan; ^2^ Cancer Prevention and Control Division, Kanagawa Cancer Center Research Institute, Yokohama, Japan; ^3^ Graduate School of Health Innovation, Kanagawa University of Human Services, Kawasaki, Japan; ^4^ Center for Innovation Policy, Kanagawa University of Human Services, Kawasaki, Japan

**Keywords:** hereditary tumor, artificial intelligence, breast cancer, germ-line cells, cancer screening

## Abstract

Personalized cancer screening guided by genomic information holds the potential for effective cancer prevention. However, current approaches face challenges, including the low risk associated with most genetic variants and limited experience in genetic counseling for healthy individuals. We are initiating a feasibility study that employs a germline cancer genomic panel of 30–80 high-risk cancer genes, such as BRCA1 and BRCA2, to identify individuals with pathogenic variants. Genetic counseling will be conducted by certified medical doctors and genetic counselors utilizing the Kanagawa Prospective “ME-BYO” Cohort Study. This initiative seeks to adapt hereditary cancer counseling practices to preventive medicine, addressing the differences in counseling between clinical and preventive settings. It aims to establish a framework for personalized cancer screening, contributing to future guidelines for identifying individuals at high risk of hereditary cancers. This research will help bridge the gap between genomic research and practical preventive medicine, paving the way for personalized cancer screening strategies based on genetic information.

## 1 Introduction

Personalized medicine guided by genomic information holds great promise for advancing cancer prevention. By elucidating the constitution defined by the human genome, we can perform personalized cancer screening based on personal genomic information. According to this strategy, intensive cancer screening, including magnetic resonance imaging or risk reduction treatments, could be provided. Currently, some examinations and treatments are already being provided for patients with hereditary cancers as standard medical practice. However, despite recent advances in genomic research, personalized cancer prevention has not been utilized widely for several reasons. First, recent advances in genomic research, such as genome-wide association studies (1), have led to the availability of direct-to-consumer genetic testing (2) for the general population. However, most identified genetic variants carry a low risk of developing cancer, and the supporting evidence is often insufficient. Consequently, the clinical utility of these findings for cancer prevention remains limited. Therefore, large genome cohort studies in Japan are being conducted to simultaneously obtain genetic and detailed environmental information and analyze their interactions (3, 4). However, a period of more than 10 years will be needed to clarify the relationship with actual cancer incidence. Moreover, practical strategies for personalized cancer prevention using genomic information remain unclear. Second, limited knowledge and practical experience hinder the effective delivery of genetic counseling to cancer-free individuals undergoing genomic testing results for preventive screening. Third, genetic testing for cancer screening of the general population is costly.

## 2 Novel initiatives for personalized cancer screenings

A framework for personalized cancer screenings has recently been established, and the cost of genomic analysis has decreased dramatically due to recent technological innovations. We focused on the germline cancer genome panel, which has recently become widely used in genomic medicine. This panel test can comprehensively analyze approximately 30–80 genes with a high risk of cancer development, such as *BRCA1* and *BRCA2*. The cost of this test is approximately 150,000–200,000 yen (about 1,000–1,300 USD) (5). Although prices have dropped considerably in recent years, it remains more expensive than other optional cancer screening methods, such as blood tests and imaging examinations, which are typically conducted annually or biennially. In contrast, because this test is performed only once in a lifetime, individual with a family history of cancer may be willing to bear the costs. Personalized measures centered on surveillance after diagnosis have been established, with clear medical benefits for those who undergo testing; individuals identified as having a genetic predisposition can opt for intensive surveillance, such as frequent magnetic resonance imaging. Those at particularly high risk, such as carriers of pathogenic variants in BRCA1 or BRCA2, may also consider risk-reducing surgeries as part of their preventive care. This is a clear difference from the direct-to-consumer genetic tests currently available on the market. However, to widely implement this approach in clinical practice, determining the optimal genetic counseling system remains a major challenge. Currently, genetic counseling, provided by specialist doctors and certified genetic counselors in Japan, is mainly targeted toward patients with cancer; moreover, physicians have limited experience in providing such counseling in the field of preventive medicine.

## 3 Barriers to implementation

Several differences exist in genetic counseling between clinical and preventive medicine. First, the target individual may be a cancer patient in clinical settings or a healthy person in preventive contexts. However, this difference can be overcome by applying genetic counseling methods being used at specialist medical institutions for unaffected relatives of patients with hereditary tumors. Second, the prior probability of hereditary cancers in patients who receive genetic counseling may differ significantly from that in patients who receive genetic counseling at cancer hospitals. Cancer hospital visitors are either patients with cancer or unaffected relatives of patients diagnosed with hereditary cancers, and the likelihood of undergoing testing is high. For example, the probability of hereditary breast-ovarian cancer has been reported to be approximately 10% (6).

Conversely, the proportion of people with pathogenic variants of cancers is much lower; the percentage of *BRCA1* or *BRAC2* pathogenic variants in the general population is reported to be 0.2–0.3% (7, 8), and even if the frequency of pathogenic variants in other genes covered by panel testing is considered, it is unlikely to be very high. Therefore, individuals are required to be targeted for testing by pre-screenings, selecting a high-risk group of pathogenic variants. For example, it is necessary to understand the National Comprehensive Cancer Network (NCCN) guidelines and conduct medical interviews with people. The guideline presents criteria for genetic testing, including individuals diagnosed with breast cancer at age 50 years or younger, and those with triple-negative breast cancer. For individuals without cancer diagnosis, testing is also recommended if they have close blood relatives with breast cancer diagnosed at age 50 years or younger, male breast cancer, ovarian cancer, or pancreatic cancer. Further, specialized medical professionals, such as genetic counselors or physicians whose subspecialty is genetic medicine, are required, which currently remains a major obstacle. However, this can be solved by utilizing an AI Chatbot system that we have recently developed (9, 10).

## 4 Design of our study

**Figure 1:**
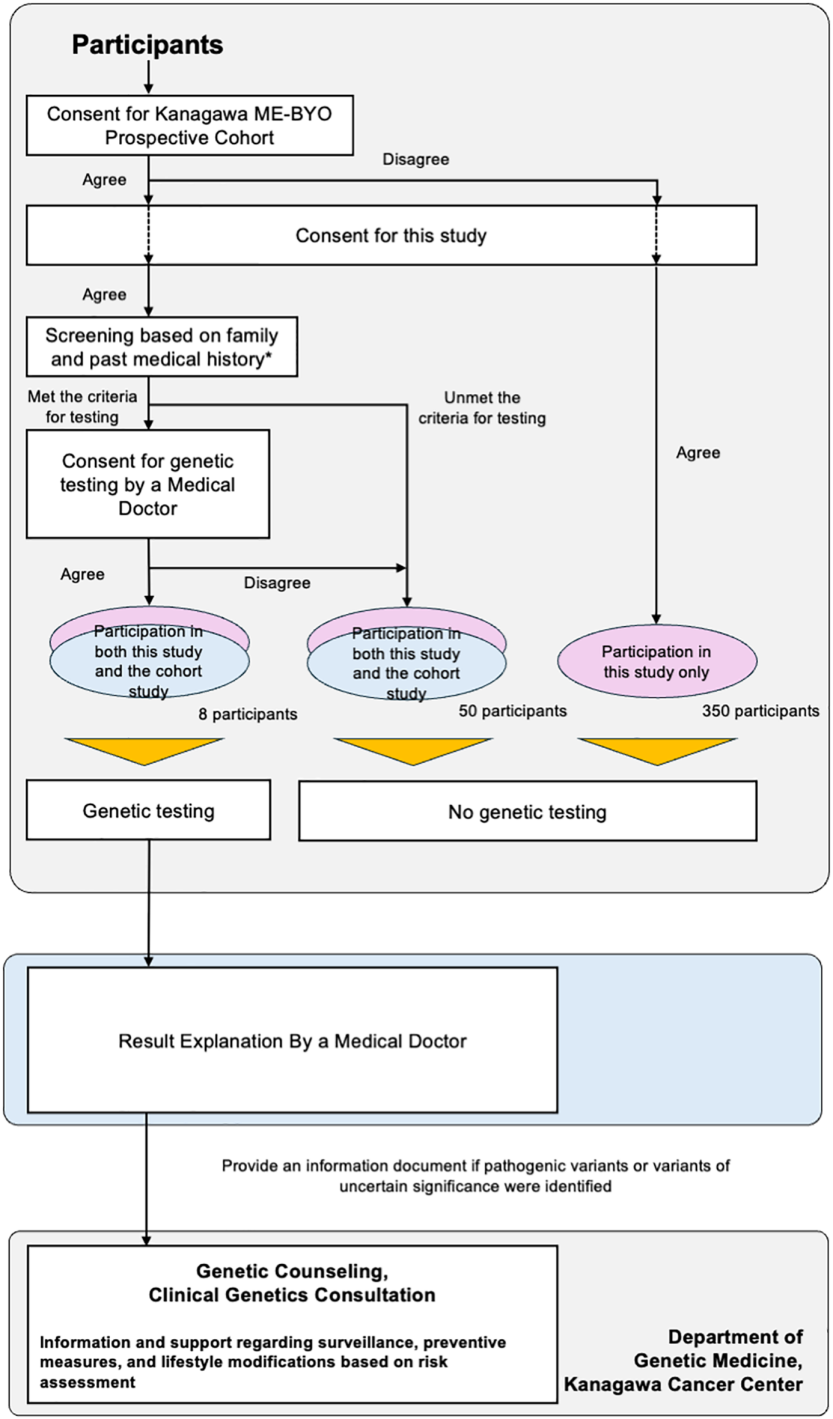
Study design of “Implementation of a Cancer Risk Assessment Program Using Genetic Testing in the General Female Population”.

We have planned to conduct a feasibility study, titled “Implementation of a Cancer Risk Assessment Program Using Genetic Testing in the General Female Population (UMIN000056304) (**Figure 1**).” This study will utilize the know-how of hereditary tumor counseling, which is routinely provided to patients with cancer at medical institutions. Further, we will use the research platform of the Kanagawa Prospective “ME-BYO” Cohort Study (the ME-BYO Cohort) (11) in this study. Participants have been enrolled in advance for this population-based cohort study. The certified medical doctors and certified genetic counselors of the Department of Genetic Medicine, Kanagawa Cancer Center, will provide genetic counseling for the participants with pathogenic variants.

## 5 Discussion

The model of the personalized cancer screening program developed in this study can be used to realize tailor-made cancer screening strategies that utilize genetic information obtained from whole-genome analysis, which will become available in the near future. The model developed in this study targets the causative genes of hereditary cancers with a high risk of cancer development.

Essential next steps include collaborative activities with other projects and institutions in Japan. In 2023, Tohoku Medical Megabank Organization announced that it had started reporting germline findings, including those related to hereditary breast and ovarian cancer and Lynch syndrome, to their study participants (12). In addition to experience in the high-volume center of genetic medicine, the accumulated experiences from population-based genomic cohort studies would greatly benefit from these collaborative activities. Finally, we can create guidelines for identifying patients at a high risk of hereditary cancers, significantly contributing to the development of personalized cancer screening.

## Data Availability

The original contributions presented in the study are included in the article/supplementary material. Further inquiries can be directed to the corresponding author.
